# Physical activity and nutrition program for seniors (PANS): protocol of a randomized controlled trial

**DOI:** 10.1186/1471-2458-10-751

**Published:** 2010-12-06

**Authors:** Linda Burke, Jonine Jancey, Peter Howat, Andy Lee, Deborah Kerr, Trevor Shilton, Andrew Hills, Annie Anderson

**Affiliations:** 1 School of Public Health, Curtin University, GPO Box U 1987, Perth, WA 6845, Australia; 2 Centre for Behavioural Research in Cancer Control, Curtin University, GPO Box U 1987, Perth, WA 6845, Australia; 3 National Heart Foundation, Western Australia Division, 334 Rokeby Road, Subiaco, WA 6008, Australia; 4 Institute of Health and Biomedical Innovation, Queensland University of Technology, 60 Musk Avenue, Kelvin Grove QLD 4001, Australia; 5 Centre for Public Health Nutrition Research, University of Dundee, Dundee, DD1 4HN, Scotland, UK

## Abstract

**Background:**

Along with reduced levels of physical activity, older Australian's mean energy consumption has increased. Now over 60% of older Australians are considered overweight or obese. This study aims to confirm if a low-cost, accessible physical activity and nutrition program can improve levels of physical activity and diet of insufficiently active 60-70 year-olds.

**Methods/Design:**

This 12-month home-based randomised controlled trial (RCT) will consist of a nutrition and physical activity intervention for insufficiently active people aged 60 to 70 years from low to medium socio-economic areas. Six-hundred participants will be recruited from the Australian Federal Electoral Role and randomly assigned to the intervention (n = 300) and control (n = 300) groups. The study is based on the Social Cognitive Theory and Precede-Proceed Model, incorporating voluntary cooperation and self-efficacy. The intervention includes a specially designed booklet that provides participants with information and encourages dietary and physical activity goal setting. The booklet will be supported by an exercise chart, calendar, bi-monthly newsletters, resistance bands and pedometers, along with phone and email contact. Data will be collected over three time points: pre-intervention, immediately post-intervention and 6-months post-study.

**Discussion:**

This trial will provide valuable information for community-based strategies to improve older adults' physical activity and dietary intake. The project will provide guidelines for appropriate sample recruitment, and the development, implementation and evaluation of a minimal intervention program, as well as information on minimising barriers to participation in similar programs.

**Trial Registration:**

Australian and New Zealand Clinical Trials Registry ACTRN12609000735257

## Background

Australia has experienced a steady increase in the proportion of older adults, with projections that 22% of the population will be aged over 60 by 2025 [1,2]. This ageing population is heavier than a generation ago, with in excess of 60% of older adults now classified as overweight or obese [3]. Overweight and obesity levels are increasing at a rapid rate worldwide while other non-communicable diseases (NCDs) such as heart disease, type 2 diabetes and cancer are also on the rise [4,5]. It has been estimated that in developed countries, the cost of obesity equates with 0.7 to 2.8% of the total yearly health expenditure [6]. For example, the annual cost of obesity is estimated to be $21 billion in Australia and $2.1 billion in the state of Western Australia [7]. The increase in the prevalence of overweight and obesity is of particular concern, in view of the strong association between excess body weight and chronic health problems. It is known that as age increases physical activity declines [8], with 46% of Australians aged 60 to 75 years being insufficiently active and 33% being completely sedentary [1,9]. Over the years, the physical activity levels of older Australians have reduced [9,10] while their food consumption has increased [11]. This follows the worldwide trend in diet which is shifting towards an increased consumption of saturated fats, with the level of fat consumed exceeding the recommended proportion of daily energy intake [12-14].

The benefits of regular physical activity are well recognised [5,15,16,18-21], regardless of body mass index (BMI) [22]. The greatest health improvements appear to occur when a person moves from being sedentary (<100 mins/week) or involved in light (1-2.9 METS) to moderate-intensity activity (>3 METS) [23]. Low-intensity aerobic exercise is typically recommended for older adults as it can be sustained for longer, results in less tiredness and injury, and therefore may result in greater energy expenditure than high-intensity exercise [24]. The Australian Government has recently developed physical activity guidelines for older Australians to help improve their health and well being. The guidelines recommend that moderate-intensity physical activity be performed for a minimum of 30 minutes on most, preferably all, days of the week [25,26]. In addition, eating adequate amounts of fruit and vegetables can provide essential nutrients for healthy tissue bolster the immune system and protect against chronic diseases [5,27-29]. The Australian Guide to Healthy Eating [30] recommends between four to seven 75 gram serves of vegetables and two to three 150 gram serves of fruit for adults aged over 60 years. In addition, both dietary fat and refined carbohydrate should be reduced to achieve appropriate balance in macronutrient intake necessary for an acceptable body weight [31].

Maintaining adequate levels of physical activity [32]and sustaining an appropriate diet [33] are important public health goals to address obesity and to minimise the adverse physiological changes [34] associated with ageing. However, there remains a need for systematic assessment of dissemination strategies to improve health outcomes [35,36], recognising that older people are a heterogeneous group that would benefit from interventions to suit their personal needs and circumstances [37]. The design of interventions needs to be rigorous [38] with large samples and longer time frames [39,40]. Additionally, home-based nutrition and physical activity programs for older adults may reduce future costs to health care [37]. This paper describes the protocol of a randomised controlled trial that aims to improve the physical activity and nutrition behaviours of insufficiently active people aged 60 to 70 years.

## Methods/Design

### Study design

This project will consist of the development, implementation and evaluation of a physical activity and nutrition intervention. The program is designed to increase physical activity levels, enhance nutritional intake and assist in the management of body weight of insufficiently active 60-70 year-olds. It will be conducted in metropolitan Perth, the capital of the State of Western Australia. The intervention and evaluation design has been based on a large pilot project that produced encouraging results with respect to adherence and behaviour change [41].

**Figure 1:**
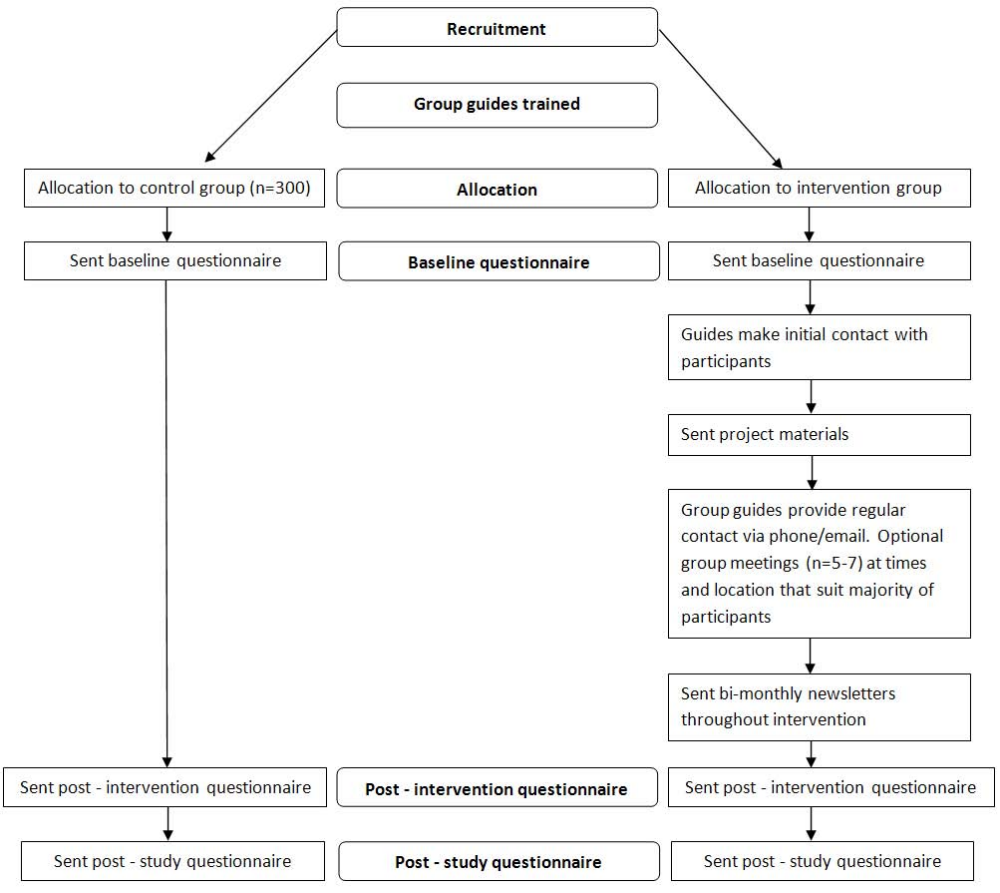
**PANS study plan**.

The study will be a 12-month randomised controlled trial (RCT) (Ref Figure 1). Data will be collected from participants over three time points at pre-intervention, immediately post-intervention and at 6-months post-study. The project protocol has been approved by the Curtin University Human Research Ethics Committee (approval number HR 186/2008).

### Recruitment

A stratified random sampling procedure will be adopted to recruit participants from 60 suburbs (neighbourhoods) within the Perth metropolitan area. Selection criteria for these suburbs are: (a) comprised of at least 14% 60 year-olds and above, reflecting the State average [42]; (b) containing at least 120 adults aged 60-70, to ensure a sample size sufficient for the matching of telephone numbers to the Perth Electronic White Pages [43]; and (c) are of low or medium socio-economic status (SES) based on the Socio-Economic Index for Area (SEIFA) [44], a value derived from income, education level, employment status and skill level. Suburbs will be arbitrarily matched for low and medium levels of socio-economic status. The suburbs will then be assigned to either the intervention group or the control group using a table of random numbers. The sample size will be n = 300 for each of the intervention and control groups at baseline. This strategy is based on a previous RCT conducted by the research group that successfully recruited a similar study sample [45].

Using the Federal Electoral Roll (FER), 7200 potential participants in total will be randomly drawn from the 60 Perth suburbs, with the aim to recruit 15 participants per suburb. Participants need to be: (a) "insufficiently active", i.e. not achieving 30 minutes of moderate-intensity physical activity on at least 5 days per week [25]; (b) aged 60 to 70 years; (c) healthy to the extent that participation in a low-stress physical activity and nutrition program would not place them at risk; (d) not to have taken part in any research studies that involve exercise or nutrition within the last five years; and (e) not to be on any special diet.

### Procedure

The Survey Research Centre at Curtin University will match telephone numbers of the 7200 names from the FER to the Perth Electronic White Pages prior to making the initial contact. It is anticipated that the matching will yield an 80% success rate [45]. During the initial contact, the purpose of the study will be explained and the caller will determine whether the individual meets the selection criteria. Participants who give verbal consent will be assigned to an intervention or control group. A self-completion questionnaire will then be sent to them, along with an explanatory cover letter and self-addressed envelope for returning the questionnaire. Participants will be advised to complete the Physical Activity Readiness Questionnaire and to furnish a medical clearance if deemed necessary before commencing the program.

### Intervention implementation strategies

#### 1. Staff training

Senior university Health Science students with expertise in physical activity, nutrition and health promotion will be recruited as "Guides". Potential Guides will undergo screening for suitability and intensive training; receive a comprehensive Guide's manual of dietary and physical activity guidelines; receive regular support via email and phone contact from the project coordinator; and be awarded a certificate upon completion of the training. The aim is for Guides to follow the successful New Zealand ‘Green Prescription Program' [21,46,47]. They will be responsible for coordinating regular group meetings; phone/email contacts with participants; and be accessible for information sharing and answering questions. They will be supervised by an accredited Dietitian and a Human Movement Specialist.

#### 2. Provision of resources and instructional materials to participants

The intervention group participants will receive a booklet designed to motivate them to improve their levels of physical activity and their nutrition, through goal setting. The booklet which has been updated from a pilot project [41,48,49]; will be supported by additional written materials including an interactive calendar and exercise chart. The intervention group will also be provided with a resistance band to perform the exercises described in the program, and a pedometer to monitor walking and to record the number of daily steps. A bi-monthly newsletter will reinforce the key messages.

#### 3. Follow-up and support

The intervention group will be allocated Guides who will conduct suburban-based group meetings (available to those who can attend) and monitor the progress of their group participants. Each Guide will supervise and support participants in one or two suburbs (n = 10 to 20). They will contact their participants via phone (or email if preferred). The Guides will make three pre-arranged motivational phone calls at 4, 12 and 20 weeks to give advice and individualised consultation, as well as to monitor attainment of goals and provide encouragement, support and feedback [46,47,50]. Guides will maintain a detailed log book of the contacts made with their allocated group of participants. To increase the likelihood of sustainability, the National Heart Foundation will provide ‘Heartline', a website and 1-800 telephone number for further information. All resources are designed to support participants adoption of health-enhancing behaviours with the opportunity to access information and have questions related to physical activity and nutrition answered.

### Control group

Requests to complete the self-administered questionnaires will be the only contact the control group will receive from the project staff.

### Outcome measures

A self-administered questionnaire will be completed by both groups of participants at baseline (pre-intervention), 6-months (immediately post-intervention) and 12-months (6-months post-intervention). The questionnaire comprises of previously validated instruments [20], and will undergo further reliability testing prior to its initial use at baseline.

Physical activity will be measured using *The International Physical Activity Questionnaire *(IPAQ) [51]. IPAQ has undergone extensive reliability and validity testing in 12 countries. The instrument has acceptable measurement properties for use in many settings and is specifically designed for population-based prevalence studies of physical activity. A strength exercise question based on recommendations from the American Heart Association [32] will be included to ensure the main components of the home-based exercise program are also measured.

Dietary intake will be measured using a modified version of the *Fat and Fibre Barometer *[52]. A question from the New South Wales Government report on soft drinks [53] will be appended to measure frequency of soft drink consumption. Validated questions will also confirm participants' stages of change regarding fruit and vegetable consumption [54]. A question from the Western Australian Physical Activity Taskforce 2005 *State-wide adult physical activity survey *will be used to assess confidence to participate in at least 30 minutes of physical activity on five or more days of the week [55].

General physical and mental health will be measured by *The Medical Outcomes Study Short-Form Health Survey *(SF-8) [56]. SF-8 is a standard generic international instrument to assess health status and is comprised of two summary scales - the physical component summary (PCS) score and the mental component summary (MCS) score.

Social support will be measured by the *Dukes Social Support Scale *(DSSI) [31]. The Scale is a subjective evaluation of the type and number of social interactions and has been validated for use with older people. The instrument contains two sub-scales that measure social interaction and satisfaction and has good internal consistency (Cronbach's alpha 0.77) and test-retest scores (0.70 to 0.81)[31].

A single item from the *Social Support for Physical Activity questionnaire *[57] (SSPA) will also be used to measure perceived levels of social support for physical activity provided by friends and family. In addition, a single question will be asked about loneliness [58] while a standard validated question [59] will be used to confirm the participants' smoking status.

Demographics will be assessed by questions on gender, age, educational level, country of birth, marital status, socioeconomic status, financial status and co-morbidities. Anthropometric measures will include self-reported height and weight, waist and hip girth. A recent study has confirmed that self-assessment measures are suitable for such studies when a correction factor is applied [60].

A sub-sample of 100 participants will be selected from the intervention group. Following self-report of their height, weight, waist and hip girths the research team will measure each of these variables. Calculations of differences between self-reported and measured data will be undertaken to identify a correction factor based on the methodology of Dhaliwal et al. [60].

### Process evaluation

A brief feedback sheet will be mailed to all participants to evaluate the booklet [48]. It invites the participants to rate the booklet in terms of interesting to read, easy to understand, usefulness of advice, suitability for the age group, and the relevance of messages. Participants will also be asked to comment on specific features they particularly like or dislike, as well as suggestions for further improvement [61]. The calendar, exercise sheet and other program resources will also be evaluated via a similar previously utilised feedback format [48].

### Sample size

This is a RCT with outcomes measured at three time points. Power calculations are based on linear mixed model and assuming 70% complete data across the three assessments due to attrition and non-respondents. In the power analyses, effect sizes of interest are associated with the correlation coefficient (or semi-partial correlation). For the mixed regression analyses of physical activity times and metabolic equivalent tasks, a sample size of n = 600 [150 per gender by intervention or control group] will provide sufficient power (80%) to detect a medium effect size (accounting for approximately 16% of the variance) for gender by age interactions at a single time point without covariate adjustment. Power to detect these same interactions in the trends (based on 3 assessments) is sufficient to detect a smaller effect, accounting for approximately 11% of the variability.

## Discussion

### Results from the PANS study are due in mid-2011

As the aging population increases there is an urgent need to develop sound interventions capable of making a positive change to health status with consequent reduction in pressure and cost to the health care system. This physical activity and nutrition program offers a unique approach compared to other such programs for older people previously conducted in Australia for the following reasons.

The target group will be selected from younger seniors groups (60-70 years), and low and medium SES groups rather than high SES groups. Samples will be randomly selected and actively recruited through the Australian FER, and not community volunteers recruited through advertising. The intervention will provide valuable data on the effectiveness of strategies to improve dietary intake and increase physical activity in the community. The project has been designed to evaluate the strength of combining both physical activity and nutrition in order to improve the health of seniors. The evaluation data will be collected from participants in their own communities and not in a research centre, making the program relevant to the normal population and not limited to a clinical group or setting. The project will provide guidelines for appropriate sample recruitment, and the development, implementation and evaluation of a minimal, home-based tailored physical activity and nutrition intervention program. The information gathered will be useful for minimising barriers to participation in physical activity and nutrition programs. The outcomes of the project will have significant potential benefits to the Australian community via increased physical activity and better nutrition to reduce chronic disease (and associated costs), as well as enhanced mental health and improved quality of life.

## Competing interests

The authors declare that they have no competing interests.

## Authors' contributions

LB coordinated the PANS program and drafted the manuscript. JJ, PH, AL, LB, TS, DK, AH and AA designed the study, and revised the manuscript. All authors read and approved the final manuscript.

## Pre-publication history

The pre-publication history for this paper can be accessed here:

http://www.biomedcentral.com/1471-2458/10/751/prepub
